# Intradiscal Steroid Injections for Degenerative Disc Disease With Modic Changes: A Retrospective Study of Therapeutic and Diagnostic Features

**DOI:** 10.7759/cureus.58333

**Published:** 2024-04-15

**Authors:** Jason L Marcus, Benjamin D Westerhaus, Jacob Fleming, Douglas P Beall, Isis Sweeney, Mark Lewis, Vidur Ghantiwala, Anthony Giuffrida

**Affiliations:** 1 Medicine, Nova Southeastern University Dr. Kiran C. Patel College of Osteopathic Medicine, Fort Lauderdale, USA; 2 Interventional Spine & Pain Management, Cantor Spine Center at the Paley Orthopedic & Spine Institute, Fort Lauderdale, USA; 3 Interventional Radiology, Comprehensive Specialty Care, Edmond, USA

**Keywords:** modic changes, discography, discogenic back pain, axial back pain, anterior column pain, chronic low back pain (clbp)

## Abstract

Purpose: Anterior column pain refers to axial low back pain (LBP) originating from the intervertebral disc or vertebral endplates (discogenic or vertebrogenic pain). We sought to assess the safety and effectiveness of intradiscal steroid injection (IDSI) in diagnosing and treating patients with LBP arising from the anterior column.

Patients and methods: This is a retrospective chart review of 66 patients who underwent 77 injections in an outpatient, private practice setting for the treatment of chronic lower back with history and physical exam findings indicating an origin within the anterior column and magnetic resonance imaging (MRI) findings of Modic changes associated with disc degeneration of grade 4 or above on the modified Pfirrmann scale. Patients reported pain as measured by the numerical rating scale (NRS) before the injection, at the time of their follow-up, and their maximum pain relief. The primary outcome was the change in NRS before and after the injections. The secondary outcome determined if the changes in the subjects’ NRS met the minimal clinically important change (MCIC) criteria for LBP. We conducted a statistical analysis using a paired sample t-test.

Results: There was a statistically significant difference between the pre-injection and follow-up NRS scores (p < 0.001) and a significant difference between pre-injection and maximum relief NRS scores (p < 0.001). Most subjects (55/77, 71.4%) met the MCIC to relieve their chronic LBP at the time of the follow-up evaluation.

Conclusion: For patients with chronic LBP and degenerative endplate changes, IDSIs provided these patients with significant short-term pain relief from pain arising from the anterior column.

## Introduction

Chronic low back pain (LBP) is one of the leading causes of disability and hospitalization in the world, affecting up to one billion people globally and 45 million people a year in the United States [1,2]. Discogenic pain has been identified as the leading cause of LBP, accounting for nearly 400 million people worldwide and 20 million people in the United States [3,4]. Treatment options for discogenic LBP range from physical therapy, chiropractic treatments, epidural injections, acupuncture, and anti-inflammatories. More invasive interventions, such as spinal fusion and intervertebral disc arthroplasty, are also considered [4]. In recent years, the clinical distinction between discogenic pain and vertebrogenic pain, as defined by anterior column pain associated with degenerative endplate changes, has become more important with the development of treatments for these sources of back pain. Discogenic pain refers to pain arising from degeneration of the intervertebral disc, disc dehydration, and fissuring of the annulus fibrosis. Vertebrogenic pain is characterized by vertebral endplate degeneration, including edematous (Modic type I), fibrofatty (Modic type II), and more pronounced erosive changes. Vertebrogenic changes are also associated with a loss of disc height and disc dehydration, making it often difficult for clinicians to distinguish between these pathologies. The classical clinical presentation for patients with these two pathologies is nearly indistinguishable. Both patient cohorts present with centralized back pain exacerbated by axial loading and spinal flexion as the primary symptoms. The term "anterior column pain" has been used to describe pain with a discogenic etiology, a vertebrogenic etiology, or a combination of the two. Intradiscal steroid injections (IDSI) to diagnose and treat anterior column LBP have been a topic of variable enthusiasm over the past 30 years.

Intradiscal steroid injections became more common in the 1990s after the original description of inflammatory changes of the vertebral endplate on magnetic resonance (MR) images by Michael Modic [5]. He described three distinct degenerative endplate changes, later known as Modic changes. Only type I and II Modic changes are recognized as potential pain generators. Modic type I changes indicate acute inflammation of the vertebral endplate caused by fissuring and fibrovascular granulation tissue. Modic type II changes arise from chronic damage with adipose marrow replacement of the endplate [6]. Type I changes have a decreased signal intensity on T1-weighted MR images and an increased signal intensity on T2-weighted MR images, including short-tau inversion recovery (STIR) and other fat-suppressed T2-weighted sequences. In contrast, Type II Modic changes are seen as increased signal intensity on T1-weighted and non-fat-suppressed T2-weighted MR imaging (MRI), with decreased signal intensity on STIR and other fat-suppressed sequences [5,6]. In conjunction with advancements in fluoroscopic-guided minimally invasive spine procedures, identifying types I and II Modic changes provided imaging evidence of potential pain generators. It paved the way for the increasing popularity of IDSIs [5,6]. The rationale behind using glucocorticoids in intradiscal injections is their anti-inflammatory properties, which, when used as an oral treatment or inserted into the disc space, were predicted to reduce or eliminate patient pain and discomfort [7]. Discography, a minimally invasive diagnostic procedure, can provide evidence as to the presence and location of discogenic pain [8]. Some studies have discouraged the use of intradiscal injections, with or without steroids, arguing that they may do more harm than good [8]. The primary criticism is that the injection site penetrates the intervertebral disc, which may result in accelerated disc degeneration. Some studies question whether the therapeutic effect outweighs the potential risk of further degeneration, though these studies have significant limitations [9,10].

In a study conducted on post-mortem subjects, steroid-injected intervertebral discs were found to have decreased bulging and increased disc height [11]. This suggests that IDSIs may have other therapeutic effects besides their anti-inflammatory features. The mechanism of action is a topic of debate among physicians who perform and study these injections, but reducing inflammatory change is the leading hypothesis [7]. A mixture of steroids (to diminish the pro-inflammatory cytokine release) and preservative-free bupivacaine or lidocaine (to act as a neuronal blockade of the nociceptive active nerves) is often used as the injectate. The rationale behind the procedure is justified by the presence of inflammatory changes, such as degenerative vertebral endplate changes or annular tears (indicated by the presence of high-intensity zones on MRI) [4,12]. When active inflammatory changes correlate with clinical symptoms, using a steroidal anti-inflammatory for treatment may be a justifiable clinical decision.

We aimed to examine the efficacy of intradiscal steroid injections performed by a single physician using retrospective data collection. We hypothesized that intradiscal steroid injections would be an effective and minimally invasive way to provide short-term pain relief. Additionally, we aimed to confirm the pain generator for patients with anterior column pain. This confirmation was based on history, physical examination findings, and MR imaging showing Modic type I or II changes and grade 4 or higher disc degeneration according to the modified Pfirrmann grading scale [13]. These criteria were discussed by Griffith et al. as a detailed classification system for lumbar disc degeneration, assessing severity through MR imaging features, including signal intensity, disc height, and disc annulus fibers distinction [13]. The eighth grade denotes increasingly severe degeneration, with grade 4 indicating preserved disc height accompanied by hyperintense nucleus pulposus signaling and indistinct annulus fibers, characteristic of degenerative changes. We also discuss the commonly perceived benefits and challenges of performing intradiscal injections and discography. We propose that IDSI is a safe procedure with a low complication rate that contributes therapeutic and diagnostic information to the patient's pain management strategy.

## Materials and methods

Chart review

**Figure 1:**
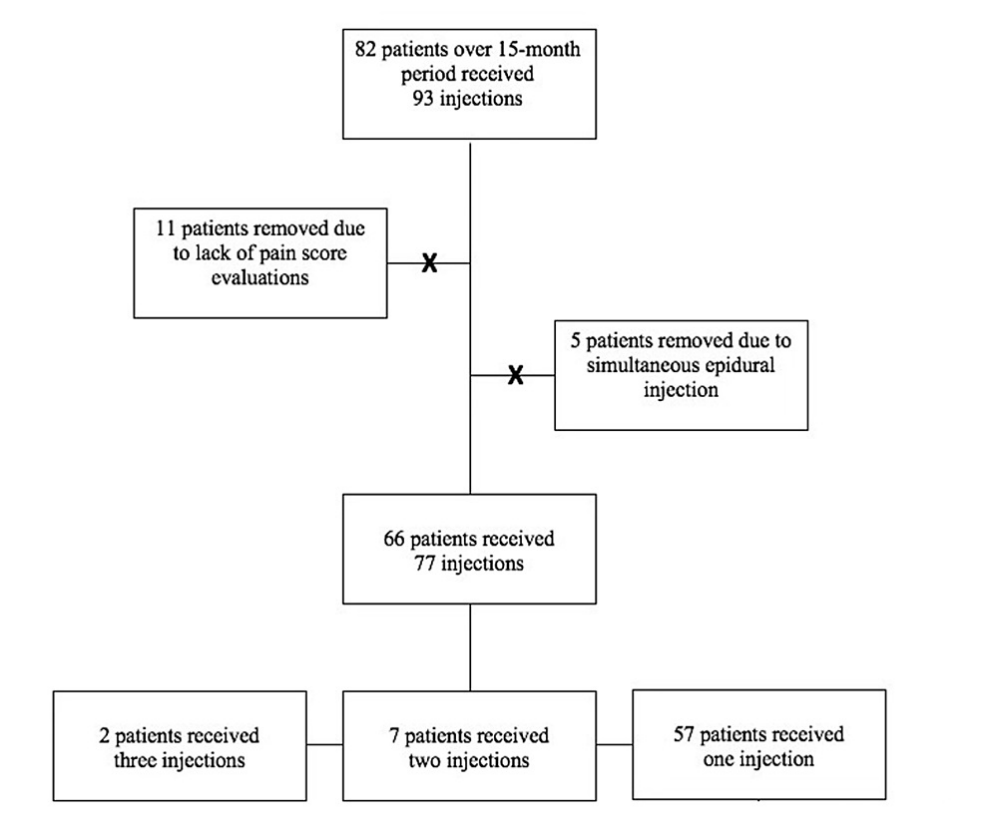
The CONSORT diagram. A visual representation and listing of the exclusion criteria of the subject population used in the retrospective analysis. CONSORT: Consolidated Standards of Reporting Trials

Institutional Review Board exemption was given to this project given the retrospective design with previously acquired patient data and the lack of direct patient identifiers. A retrospective chart review was conducted of patients within a single practice who presented with LBP and subsequently underwent IDSI over 15 months between June 2019 and August 2020. Over this time, 93 IDSI procedures were performed (Figure 1).

**Figure 2:**
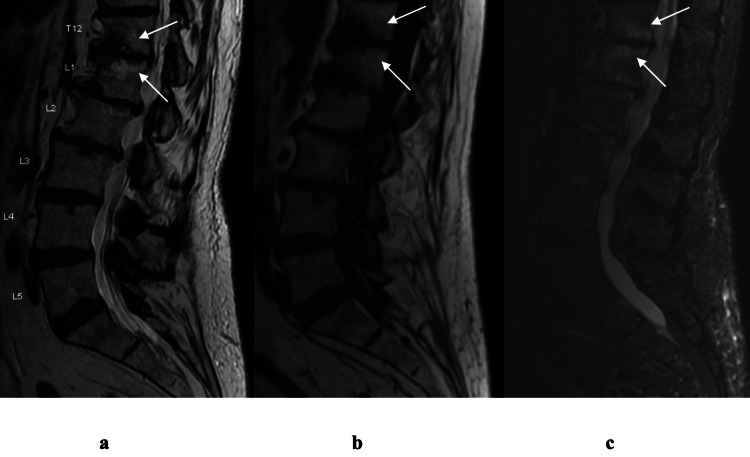
T2-weighted (a), T1-weighted (b), and STIR (c) MR images of a patient with degenerative end-plate changes with inflammation (Modic type I changes) at the T12–L1 level (white arrows in a, b, and c).

Inclusion criteria consisted of non-radiating, midline, axial low back pain possibly exacerbated by lumbar flexion or sitting (suggestive of anterior column etiology), failure of conservative treatment for at least six weeks prior to interventional treatment, and MR imaging within one year prior to the procedure demonstrating type I or type II Modic changes and modified Pfirrmann grade 4 or higher disc degeneration (Figure 2).

Each subject provided a pre-injection pain score on the 11-point numerical rating scale (NRS), a post-injection pain score, and a maximum relief pain score. The mean time to follow-up from the procedure was 19.4 days. The time of maximum relief varied for each patient and was not compiled for this study. Patients without an NRS pre-injection, NRS post-injection, follow-up appointment, or follow-up phone call (11 subjects) were excluded from the study (Figure 1).

Subjects who underwent an epidural steroid injection in conjunction with an IDSI (five subjects) were excluded from the study (Figure 1). Patients were excluded if there was a failure to obtain a post-injection pain score or if a patient received a concurrent injection other than an IDSI (Figure 1). There were 77 single-level or multilevel IDSIs (T12/L1 to L5/S1) that were performed on 66 total patients. Seven of these patients underwent two IDSIs on separate occasions, and two patients underwent three IDSIs on separate occasions (Table 1).

**Table 1:** Patients’ descriptive profile. Categorical variables such as gender and injection number were represented by frequency counts (n) and percentages (%), whereas continuous variables such as age were denoted by mean values accompanied by their respective ± standard deviations.

Variables	n (%)/Mean±SD
Age	62.4±17.3
Gender	
Male	51 (66.2)
Female	26 (33.7)
Number of Injections	
One	57 (86.4)
Two	7 (10.1)
Three	2 (3.03)

Out of a total of 93 IDSI procedures, 77 met these criteria and were used in our statistical analysis (Figure 1). Patients ranged in age from 17 to 95 with a mean age of 62, with 51 males and 26 females (Table 1).

Injection procedure

**Figure 3:**
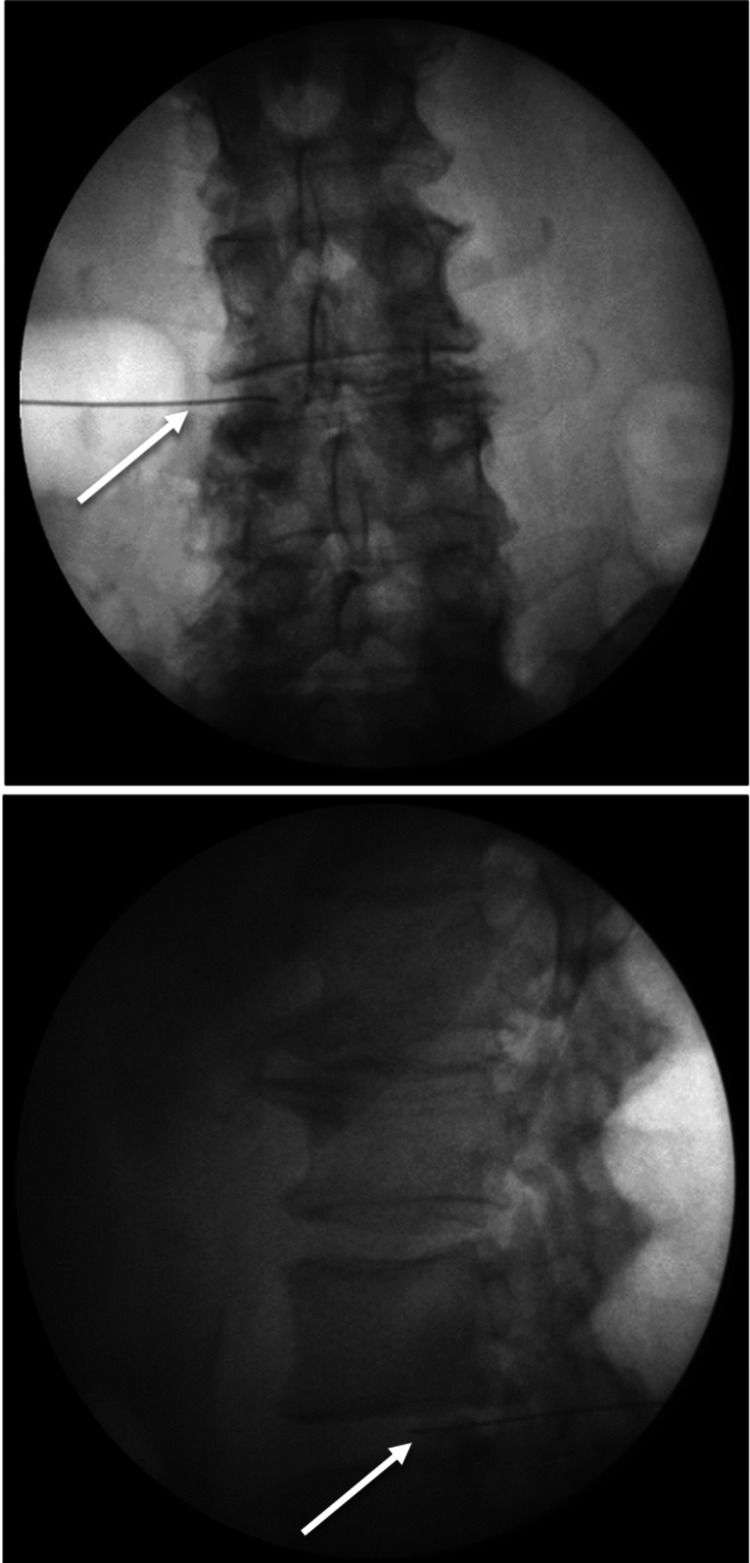
Anteroposterior (top) and lateral (bottom) fluoroscopic images of a spinal needle (white arrows) within the L3-4 intervertebral disc during an IDSI.

A single physician performed each intradiscal injection using the posterolateral disc access approach, as discussed by Furman et al. [14]. Patients were placed prone and had their skin prepped with a solution of chlorhexidine and alcohol. Patients were then given intravenous antibiotics with 2 g of cefazolin or 300 mg of clindamycin in case of penicillin allergy. The intervertebral disc was identified via fluoroscopy with an ipsilateral posterolateral oblique projection, and the overlying soft tissue was anesthetized with 1% lidocaine. A 22 gauge spinal needle was then advanced towards the center of the affected disc through Kambin’s triangle, using a right or left posterolateral oblique approach under intermittent fluoroscopy. As the disc was entered, anteroposterior and lateral visualization was used to ensure proper needle tip placement within the nucleus pulposus (Figure 3).

Once in the nucleus pulposus, 0.25 mL of iopamidol contrast was injected. Next, 0.25 mL antibiotic (1 g/10 mL cefazolin or 300 mg/10 mL clindamycin, selected based on the patient’s allergies according to consensus guidelines [15]) was injected, followed by a mixture of 1.0 mL of dexamethasone (10 mg/mL) with 1.0 mL of preservative-free 0.75% bupivacaine hydrochloride. As this mixture was injected, the patient could confirm if the fluid pressure in the disc reproduced their pain. Each degenerated disc was treated with this technique. No control disc was used in any of the procedures to avoid unnecessary injection of a normal disc.

Statistical analysis

A paired sample t-test was conducted using IBM SPSS Statistics for Mac OS (version 27; IBM Corp., Armonk, NY) to compare NRS at pre-injection baseline and follow-up, as well as NRS at baseline and point of maximal relief. For all analyses, statistical significance was set at p < 0.05.

## Results

**Figure 4:**
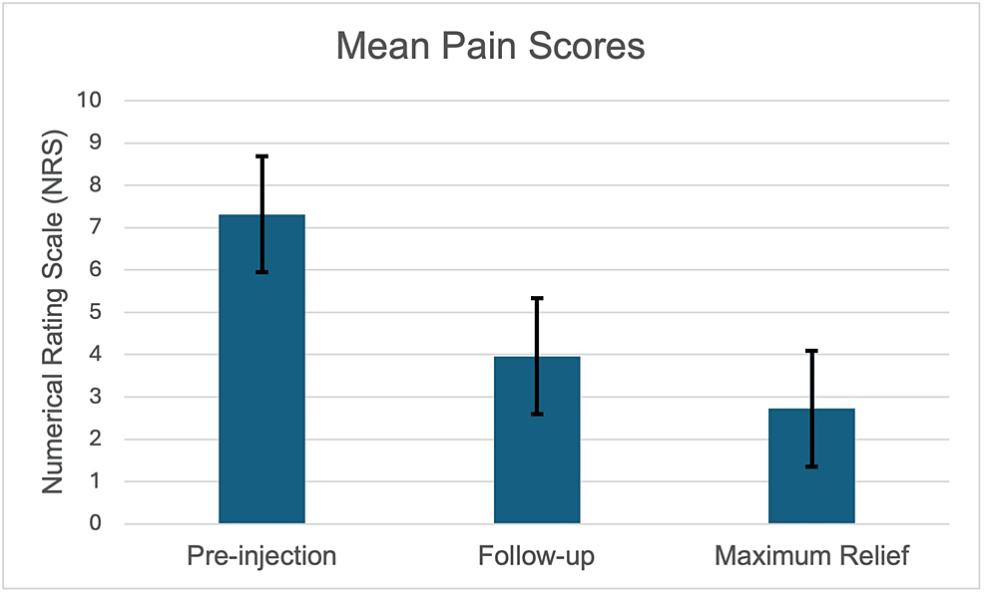
Graphic representation of the mean pain score as measured on 11-point NRS ± SD is compared before IDSI, at the follow-up appointment, and to the maximum relief achieved.

There was a statistically significant difference in the pain scores between pre-injection (7.31 ± 2.23) and follow-up evaluation (3.96 ± 2.50; t (76) = 9.47; p < 0.001). The difference in NRS between pre-injection and follow-up conditions (3.35 ± 3.11) is above the commonly accepted minimal clinically important change (MCIC) of 2.5 for patients suffering from chronic LBP (Table 2 and Figure 4) [16].

**Table 2:** Mean pain score as measured on 11-point NRS ± SD is compared before IDSI, at the follow-up appointment, and to the maximum relief achieved. Injections were performed on 66 patients. *p < 0.001 as determined by two paired t-tests. **Meets MCIC for chronic LBP.

N=77	Pre-injection	Follow-up	Maximum relief
Mean NRS (SD)	7.31 (2.23)	3.96* (2.50)	2.72* (2.36)
Δ in NRS (SD) from pre-injection	-	3.35** (3.11)	4.49** (2.92)

There was also a significant difference in the pain scores between pre-injection (7.31 ± 2.23) and point of maximum relief (2.72 ± 2.36; t (76) = 13.78; p < 0.001). The difference in NRS between pre-injection and maximum relief conditions (4.49 ± 2.92) is also above the commonly accepted MCIC of 3.5 for acute LBP and 2.5 for patients suffering chronic LBP (Table 2 and Figure 4) [16].

At the follow-up evaluation, 55 patients (71.4%) met the criteria for the MCIC of chronic LBP, and 62 patients (80.5%) met these criteria at the maximum relief evaluation (Table 3).

**Table 3:** Number of patients (n) and percentage (%) of patients who achieved minimal clinically important change (MCIC) on the numerical rating scale (NRS) during the follow-up visit and at the point of maximum relief.

Variables	n (%)
Follow-up NRS	
Met criteria for MCIC	55 (71.4)
Did not meet criteria for MCIC	22 (28.6)
Maximum relief NRS	
Met criteria for MCIC	62 (80.5)
Did not meet criteria for MCIC	15 (19.5)

Mean pre-injection and follow-up NRS met the criteria for an MCIC of greater than 30% relief, with an NRS difference of 45.8% relief. There were no cases of infection, hematoma, injection-related nerve injury, intravascular uptake, epidural spread, or subsequent compression fracture in follow-up with any of the 77 injections.

## Discussion

We propose that IDSI is a safe, low-risk treatment option to provide short-term pain relief for those suffering from anterior column LBP, and IDSI also has the additional diagnostic value of correctly identifying the patient’s pain generator. In the 77 procedures, the majority (71.4%) of patients met the criteria for the MCIC for chronic LBP at the time of follow-up. Our analysis yielded statistically significant results when comparing the pre-injection and post-injection conditions, as well as between the pre-injection and maximum relief conditions. Additionally, our mean NRS differences between the conditions surpassed the commonly accepted MCIC for chronic LBP [16]. There were no adverse events related to the procedures.

Favorable intradiscal evidence

In 2004, Butterman et al. compared the outcomes of IDSIs on 40 patients with Modic changes to 46 patients without degenerative endplate changes. At three months postoperatively, the majority (67.5%) of patients from the former group had positive opinions of the procedure with statistically significantly decreased pain scores at three months, 12 months, and 24 months compared to the non-Modic changes group who underwent an IDSI [17]. These data support the evidence that Modic changes on MRI are accurate predictors of anterior column pain and that steroid injections may provide these patients with pain relief [18].

Schellhas et al. examined patients who had active annular tears diagnosed by high-intensity zones in the posterior annulus fibrosus on MR imaging [19]. Researchers anecdotally noted that patients who failed to respond to epidural steroid injections “improved substantially with intradiscal steroid-local anesthetic injections” [19]. Patient populations that fail to respond to epidural steroid injections but subsequently respond well to intradiscal procedures further support the use of IDSI as a viable treatment option for those patients with anterior column pain. Furthermore, the additional information confirming the patient’s pain generator is important for guiding additional treatment strategies.

Unfavorable intradiscal evidence

Most concerns with IDSI center on its risk-benefit profile. Due to the varying evidence of therapeutic effect, there is concern that potential complications from injecting the intervertebral disc, including further disc degeneration, exacerbated pain, or other procedure-related injuries, would overshadow the potential benefits of using IDSI in clinical practice [9,10]. In a small, double-blind clinical trial, researchers divided 25 participants into two groups: patients receiving intradiscal methylprednisolone and those receiving intradiscal bupivacaine [10]. Simmons et al. concluded there was no significant short-term benefit in patients receiving the steroid injection, with more participants in the bupivacaine group reporting pain improvement in one of the objective pain measurements (65% versus 36% in the steroid group) but at only 10-14 days following the injection.

In a widely cited 2009 study by Carragee et al., subjects were regularly evaluated after undergoing discography and compared to a matched cohort group who had not had discography [8]. Researchers compared the lumbar and lumbosacral discs of subjects on MR imaging prior to discography and to a 7-10-year follow-up MRI, as the stated aim was to determine if subjects who underwent discography at L3/4, L4/5, and L5/S1 had further degeneration than their matched counterparts who did not undergo the procedure. The study reported that subjects who underwent discography had a “greater progression of new degenerative findings” when compared to the control subjects [9]. This study suggested that discography and, by extension, IDSI may be harmful to the intervertebral disc. However, the study excluded individuals who were appropriate discography candidates, such as individuals who had low back pain, were taking medications for back pain and had restrictions due to this pain, so the generalizability to a wider patient population is limited. Additionally, the control cohort had a far lower rate of Modic changes (11%) than the reported rates in the general population (36%). This dichotomy and a substantial loss to follow-up increases the possibility of the presence of inappropriate intergroup differences. Additionally, a follow-up of 7-10 years is subject to many environmental and experiential factors that can have deleterious effects on the disc. Importantly, the therapeutic features of intradiscal intervention to treat low back pain were not evaluated by Carragee et al. Instead, they used a diagnostic procedure that involved the pressurized injection of contrast into the intervertebral disc of sedated patients to assess the patient’s pain sensitivity without the use of a therapeutic agent. This contrasts with the methodology used in our study, where a steroid mixture was injected into a non-sedated patient with MR imaging evidence of degenerative disc disease with degenerative endplate changes who is able to give immediate feedback on their level of pain level and whether the injection mimicked their typical pain during the procedure.

Limitations of previous studies

Notwithstanding the short-term data that steroid injections may be inadequate in the treatment of discogenic pain, some researchers acknowledge that IDSIs have therapeutic benefits for appropriately selected patients [7]. We propose that the inadequacy in certain patients may be derived from the improper use of IDSI. We recommend that subjects with minimal or very minimal disc degeneration do not undergo discography or intradiscal steroid injection unless there is compelling imaging and clinical information supporting this treatment plan. We would like to emphasize the importance of proper patient screening and recommend that patients who are asymptomatic or minimally symptomatic for low back pain or discogenic pain not undergo such a procedure. Carragee et al. provided evidence that, under these circumstances, patients may have worse long-term outcomes [8]. We attribute a portion of our patients’ successes with this procedure to proper screening during the history, physical exam, and imaging evaluation completed at initial visits, with MR imaging showing evidence of degenerative disc disease with Modic changes and symptoms and physical exam consistent with non-radicular anterior column pain.

One early randomized control study of IDSIs, conducted by Simmons et al., found no evidence to suggest that IDSIs are an effective treatment for LBP. However, this study was limited by its small sample size of 25 patients, very short-term follow-up of two weeks or less, and generalized inclusion criteria for all participants. Patients receiving steroids or anesthetics included those with disc degeneration of an unspecified degree or "nonsequestered" disc herniations with a positive pain response to discography [10]. Additionally, radicular pain was not an exclusion criterion for this study, and the authors did not report the number of participants with axial LBP versus radicular pain. In a more recent and larger controlled study including patients with Modic type-I changes, the subjects responded favorably at the one-month follow-up but not at 12 months. Again, in this study, radicular pain was not an exclusion criterion, and the degree of disc degeneration was not mentioned.

As evidenced by current literature, patients with clearly defined and painful pathology, including but not limited to Modic changes, may respond favorably to IDSI [12,20]. There is little to no evidence, however, that subjects with more severe pathology respond proportionately better with more optimal post-injection outcomes. This was demonstrated by Fayad et al., who reported no difference in LBP between patients with Modic I and Modic I-II changes [12]. Notwithstanding the categorization of patients with Modic I and Modic II changes, each reported statistically significantly decreased pain one month after the IDSI but a non-significant decrease in pain at three and six months when compared to the pre-injection pain group. Zhuang et al. [21] reported similar results with decreased pain in subjects with Modic changes, with significant changes in LBP lasting into the three- to six-month period. We conclude that degenerative endplate changes associated with LBP may provide the best evidence for patients who will respond favorably to IDSI.

Identifying the benefits of IDSI

Even when patients do not receive pain relief from IDSI, the procedure still provides valuable diagnostic information as to the patient’s pain generator. If patients did not experience reduced pain, then anterior column pain may be ruled out as the primary pain generator. Alternatively, their pain may be attributed to concurrent factors such as facetogenic pain or myofascial-related back pain. This can be added to the patient’s clinical information that provides clinicians with pertinent information to determine subsequent treatment options.

Patients who have long-term relief from IDSI can potentially avoid or delay extensive surgery and the risks and adjacent segment disease that eventually accompanies a fusion procedure. Patients who undergo surgery are at higher risk for adverse events, such as nerve injury, incidental durotomy, and lengthy rehabilitation, with much higher costs while still facing the possibility of insignificant relief from their LBP [21,22]. Despite these surgical disadvantages, patients with extreme pain who receive short-term relief from IDSI may be qualified candidates for surgical intervention if alternative treatments are ineffective. Ohtori et al. found that, in patients who received therapeutic discography and had pain relief from the intradiscal injections, individuals had significant improvement in pain after surgery compared to those who underwent minimal treatment alone [23]. In addition to the therapeutic benefits, we have outlined how IDSI can diagnose the source of LBP, thereby helping surgeons make informed decisions regarding when and where to employ fusion surgery to produce the most optimal surgical outcomes [24].

Those patients who respond favorably to IDSI may be able to begin or return to conservative treatment regimens such as physical therapy (PT) or strength training, which have been shown to provide moderate relief for patients with chronic LBP [25]. This treatment combination has the potential to maximally perpetuate their pain relief and to delay the initiation of more invasive treatment options. Evidence suggests that patients with LBP may have a lower incidence of recurrence of their pain by employing these conservative measures [26]. Additionally, patients with initial positive responses to IDSIs that have recurrent pain are candidates to repeat the IDSI procedure for an additive therapeutic effect.

Finally, there has been substantial evidence suggesting that some supposed discogenic pain may, in fact, be the result of endplate inflammation and, therefore, vertebrogenic in nature. This evidence suggests that disc degeneration causes erosions and fissuring of the vertebral endplate [6]. Active pain receptors and neural branching of the basivertebral nerve (BVN) may be the neural transmission pathway of pain for those patients suffering from endplate inflammation [27,28]. A basivertebral nerve ablation (BVNA) device that has FDA clearance for use in vertebral bodies from L3 to S1 is intended for chronic LBP patients who have failed six months of conservative treatment and have Modic type I or II changes [29]. Patients who meet these criteria and have received adequate relief from IDSI are potentially ideal candidates for BVNA [6,27,28]. Relief of axial LBP after IDSI is an additional validation of anterior column pain. Regardless of the duration of pain, relief from IDSI provides meaningful information to the provider that vertebrogenic back pain is present, and BVNA to denervate this pain pathway appears to be an effective and durable treatment [30]. Lastly, although BVNA has been shown to be effective, it is more invasive than IDSI. The ablation procedure requires deep sedation (i.e. monitored anesthetic care) or general anesthesia and is accompanied by increased cost and recovery time relative to injection. IDSIs can be useful as a first-line treatment to avoid a more invasive procedure. In addition, patients who are awaiting insurance approval for BVNA may elect to undergo IDSI for temporary relief, which may be included in the practitioner's protocol to identify anterior column pain before undergoing BVNA.

There are several limitations to our study. It is a retrospective analysis where we collected medical records from a single physician and did not control for various factors including, but not limited to, concurrent facet joint edema, the use of anti-inflammatory medications, or other ongoing conservative treatment. Our analysis did not include a random sample population, as all treated subjects initially presented with LBP, and no control treatment measures were utilized. There were no reported infections, but all patients were prophylactically treated with antibiotics, which is consistent with commonly accepted treatment guidelines on intradiscal procedures.

## Conclusions

Intradiscal steroid injections may be a viable treatment option for patients with degenerative disc disease and degenerative endplate changes who are suffering from anterior column LBP. This procedure may also provide important diagnostic information in accurately identifying the patient’s pain generator. Contrary to some previously reported concerns for adverse events associated with IDSIs, we observed none within our patient cohort.
